# Piezoelectric enhancement under negative pressure

**DOI:** 10.1038/ncomms12136

**Published:** 2016-07-11

**Authors:** Alexander Kvasov, Leo J. McGilly, Jin Wang, Zhiyong Shi, Cosmin S. Sandu, Tomas Sluka, Alexander K. Tagantsev, Nava Setter

**Affiliations:** 1 Ceramics Laboratory, Swiss Federal Institute of Technology (EPFL), 1015 Lausanne, Switzerland; 2 Division of Energy and Environment, Graduate School at Shenzhen, Tsinghua University, Shenzhen 518055, China; 3 3D-OXIDES, 70 rue Gustave Eiffel, 01630 Saint Genis Pouilly, France

## Abstract

Enhancement of ferroelectric properties, both spontaneous polarization and Curie temperature under negative pressure had been predicted in the past from first principles and recently confirmed experimentally. In contrast, piezoelectric properties are expected to increase by positive pressure, through polarization rotation. Here we investigate the piezoelectric response of the classical PbTiO_3_, Pb(Zr,Ti)O_3_ and BaTiO_3_ perovskite ferroelectrics under negative pressure from first principles and find significant enhancement. Piezoelectric response is then tested experimentally on free-standing PbTiO_3_ and Pb(Zr,Ti)O_3_ nanowires under self-sustained negative pressure, confirming the theoretical prediction. Numerical simulations verify that negative pressure in nanowires is the origin of the enhanced electromechanical properties. The results may be useful in the development of highly performing piezoelectrics, including lead-free ones.

## Abstract (web summary)

Recently, negative pressure has been observed in perovskite nanowires by annealing the wires from a lower-density phase. Here, the authors show that the negative pressure enhances the piezoelectric coefficient of PbTiO_3_ and Pb(Zr,Ti)O_3_ nanowires experimentally and by *ab initio* calculations.

## Graphical abstract

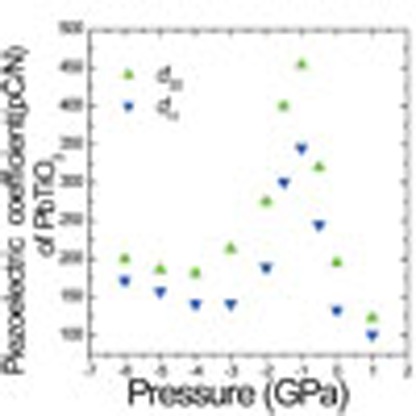

Enhanced performance of materials is one of the cornerstones of materials engineering. Such enhancements are often obtained by forming known materials in new ways that allow exceptional properties to emerge. The field of piezoelectrics is rich in examples of strong enhancement of piezoelectric properties by ingenuity in materials engineering, such as the composite approach—combining hard piezoelectric elements with soft polymers^1^, or so-called ‘domain engineering'—poling the piezoelectric crystal off its polar axis^2^.

Strain engineering has been often utilized to obtain enhancements in ferroelectric properties. Thus, enhanced *T*_c_ and *P*_s_, the Curie temperature and the spontaneous polarization, respectively, have been obtained in two-dimensional (2D) structures such as strained ultra-thin ferroelectric films^3^. In three dimensions, it was shown from first principles that negative pressure would enhance *P*_s_ and *T*_c_ in several ferroelectric perovskites^4^,^5^, the most abundant materials used for ferroelectric applications. The recent fabrication of freestanding ferroelectric nanowires of lead titanate, PbTiO_3_ (PTO) under negative pressure allowed experimental demonstration of the expected enhancements^6^.

The effect of pressure on the piezoelectric response has been well studied both from first principles^7^ and experimentally in a pressure cell^8^. It has been shown that the piezoelectric response is enhanced under positive pressure (compression); this is due to the approaching morphotropic boundary, which flattens the free-energy surface, facilitating rotational instability of the polarization under compression. The effect of negative pressure on the piezoelectric response has not been studied to date. The theoretical prediction that negative pressure drives the system through the vicinity of a different phase transition suggests that negative pressure could similarly enhance piezoelectricity.

In this work, we report on the negative-pressure effect on piezoelectricity. We show that negative pressure enhances piezoelectric response in ferroelectric perovskites with *ab initio* calculations. The positive effect of negative pressure on piezoelectric properties is also investigated and witnessed experimentally on PTO and Pb(Zr,Ti)O_3_ (PZT) nanowires derived from their lower-density phases.

## Results

### *Ab initio* study of piezoelectricity under negative pressure

We examined by *ab initio* calculations the evolution of electromechanical properties of three common tetragonal perovskite materials, PTO, BaTiO_3_ (BTO) and a highly tetragonal composition of PZT under negative pressure. To get a comprehensive portrait of these properties, we calculated the pressure dependence of the spontaneous polarization *P*_s_, the *c*/*a* ratio (*a* and *c* are the small and large tetragonal lattice constants), and the hydrostatic and longitudinal piezoelectric coefficients.

The computation of pressure dependence of *P*_s_ and *c*/*a* ratio for PTO and BTO was earlier done by Tinte *et al.*^4^ using the local density approximation, which is known to underestimate tetragonality. We followed their work, adding a similar calculation for PZT, while updating their results by using modern pseudopotentials (see Methods) within the generalized gradient approximation. The longitudinal piezoelectric coefficient *d*_33_, which is the main object of our study, was calculated in the same framework.

For a given value of hydrostatic pressure, the longitudinal piezoelectric coefficient *d*_33_=*∂P*_s_/*∂σ*_3_, where *σ*_3_ is a stress component in the Voight notation with the OX_3_ axis being parallel to the polar axis, was calculated by applying additional small stress *δσ*_3_(*δ* is to denote a difference in the variable following it) and calculating the variation of polarization *δP*_s_. The hydrostatic piezoelectric coefficient, *d*_H_=*d*_33_+2*d*_31_, was obtained as a derivative of dependence of spontaneous polarization with respect to applied hydrostatic pressure.

**Figure 1:**
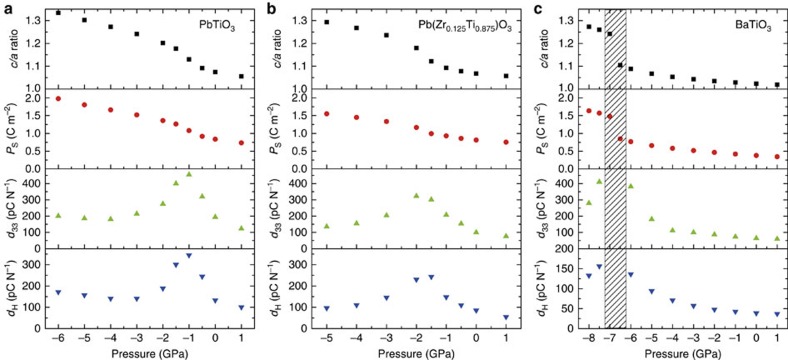
*Ab initio* calculations of effects of negative pressure in three common tetragonal perovskite materials. *c*/*a* ratio, spontaneous polarization (*P*_s_), longitudinal piezoelectric coefficient (*d*_33_) and hydrostatic piezoelectric coefficient (*d*_H_) dependence on the negative hydrostatic pressure in (**a**) PbTiO_3_, (**b**) Pb(Zr_0.125_Ti_0.875_)O_3_ and (**c**) BaTiO_3_. The hatched strip in **c** corresponds to the region of the isomorphic phase transition, where *d*_33_ and *d*_H_ diverge.

Figure 1 shows the evolution of the *c*/*a* ratio, spontaneous polarization and longitudinal and hydrostatic piezoelectric coefficient depending on the negative hydrostatic pressure for PTO, PZT and BTO. With the updated calculations, we observed a similar trend in the behaviour of the negative-pressure dependences of the material parameters (volume, strain, atomic displacements, polarization and so on) as that found by Tinte *et al.*^4^; however, the critical pressure of the transition to the supertetragonal phase in PTO and BTO is found to be smaller (in absolute value) than that calculated by Tinte. In PTO the value is ∼1 GPa, in comparison with ∼5 GPa found earlier. In BTO the value is ∼7 GPa in comparison to ∼10 GPa found by Tinte *et al.*^4^. PZT with Ti/Zr equal to 12.5/87.5 (PZT 12.5:87.5) shows the fingerprints of the supertetragonal transition too, appearing in this case at ∼1.5 GPa. It is clear that though the presence of *d*_33_ peak is independent of the *ab initio* model, the position of this maximum appears to be very sensitive to it. This may lead to additional errors in the values of the evaluated parameters. For example, in the case of PTO, the value of *d*_33_ we calculated at zero pressure is appreciably larger than the experimental one. This is a result of the rather small negative pressure for the transition to the super-tetragonal phase (∼−1 GPa), having a tail of the *d*_33_ peak that is spuriously large at zero pressure.

The transition to the supertetragonal phase can be seen (Fig. 1) by the increase in *c*/*a* and *P*_s_ in all three studied materials. It is accompanied by steep enhancement of piezoelectric coefficients. The cases of PTO and PZT are qualitatively different from that of BTO. In the case of PTO and PZT no abrupt anomalies are seen. Thus, the systems do not exhibit a phase transition in the strict thermodynamics of the term, while the smooth anomalies can be interpreted as crossing the overcritical trace of a critical point, as suggested by Tinte *et al.*^4^. In the case of BTO, our results correspond to an isomorphic first-order phase transition between two ferroelectric phases. Remarkably, for slightly different parameters of the simulation, Tinte *et al.*^4^ found overcritical trace behaviour for BTO similar to PTO. This shows that under negative pressure BTO passes very close to a critical point, thus suggesting the same origin of piezoelectricity enhancement under negative pressure in all three studied materials.

### Preparation of nanowires with build-in negative pressure

**Figure 2:**
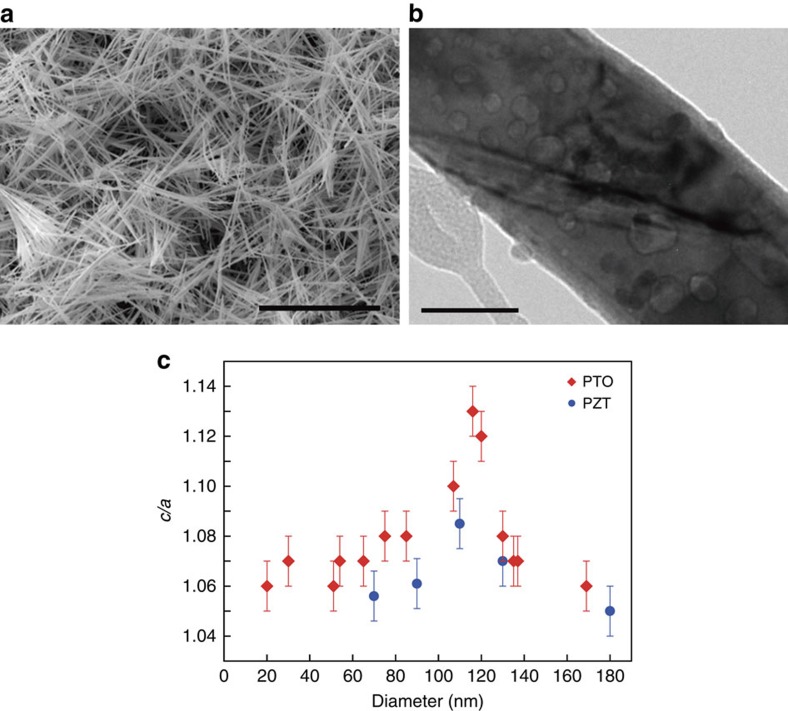
Characteristics of the studied wires. (**a**) Scanning electron microscopy image of PZT perovskite nanowires. Scale bar, 50 μm. (**b**) High-magnification transmission electron microscopy image of PZT nanowire where nanopores and domain walls are seen. Scale bar, 50 nm. (**c**) Measured tetragonality (*c/a*) in PZT and PTO nanowires as a function of wire diameter. The error bars are the maximum of deviations in *c*/*a* values measured over 3–4 regions on given wires. The data of PTO nanowires are reconstructed from ref. 6.

To obtain the material under negative pressure, we followed the methodology presented in ref. 6 for PTO, extending the range to a highly tetragonal composition of PZT. Pb(Zr_0.13_Ti_0.87_)O_3_ (PZT 13:87) was prepared hydrothermally in its PX phase^9^,^10^ and then converted to the perovskite phase by annealing in air at 600 °C for 180 min, resulting in ferroelectric nanowires of 20–500 nm diameter and up to several microns length (Fig. 2a). This processing route entails build-up of negative pressure in the wires by virtue of an inward phase transformation from a lower-density phase^6^. The wires are tetragonal and monocrystalline. Nanopores, which accompany the negative-pressure creation^6^, exist in both compositions, as shown in Fig. 2b for the case of PZT 13:87. Ferroelastic domain walls are often present (Fig. 2b). The orientation of the polar axis is random relative to the longitudinal axis of the nanowire^6^. The pressure in the nanowires increases as a function of wire diameter, with maximum pressure in PTO wires having diameters in the range ≈100–120 nm (ref. 6). At this diameter, the tetragonality is enhanced with *c*/*a* reaching 1.13, while the nominal value for PTO is 1.06 (ref. 6). *c*/*a* falls back as the diameter increases beyond 100–120 nm, due to failure in withstanding the large tensile stress as witnessed by occurrence of cracks and bigger nanopores^6^. A similar trend of the tetragonality of PZT 13:87 nanowires (measured by selected area electron diffraction and high-resolution transmission electron microscopy), with a peak of *c*/*a* also at 100–120 nm, is shown in Fig. 2c, where the tetragonality of PTO wires (extracted from ref. 6) is given for comparison.

### Experimental study on PTO and PZT nanowires

**Figure 3:**
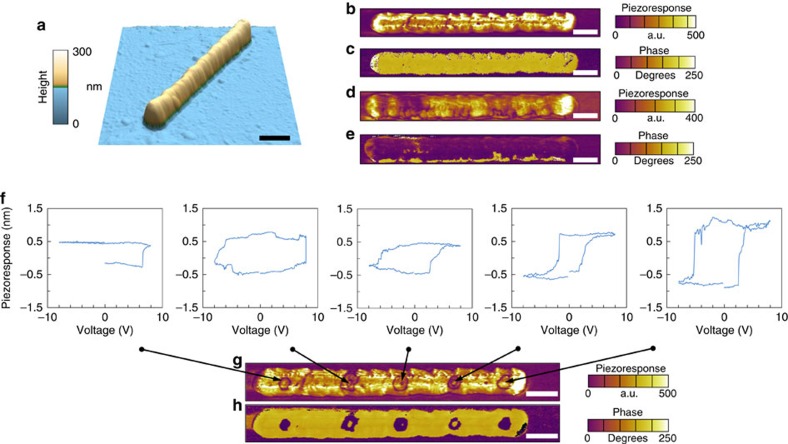
PFM analysis and switching behaviour of individual PTO nanowires. Topography image of a PTO nanowire (**a**) and native domain structure as determined from PFM amplitude (**b**) and phase (**c**) images. Amplitude (**d**) and phase (**e**) images of the same nanowire after switching with +6 V applied to the tip while scanning. Hysteresis loops (**f**) obtained from the locations indicated by the arrows to the PFM amplitude image in **g** and phase image (**h**), which shows the partial switching resulting after loop measurement. Scale bar in all images, 200 nm.

Piezoresponse force microscopy (PFM) was used to assess ferroelectricity and piezoelectric activity as a function of nanowire size. PFM was able to determine the native domain state within wires and subsequently confirm polarization switching as seen in Fig. 3 for the PTO nanowires. In addition, hysteresis loops were obtained from several locations giving information on coercive biases. Similar results were obtained on the PZT nanowires as can be seen in Supplementary Fig. 1.

The next goal was to determine the piezoelectric response (piezoresponse amplitude data) as a function of the nanowire size. PFM is suitable in this regard, as it allows for precise positioning of a nanoscale electric contact to the nanowires. In addition, nanowire size/diameter can be determined simultaneously. Accurate measurement of piezoelectric coefficients by PFM is challenging. However, several published works have presented values for effective *d*_33_ coefficients^11^,^12^,^13^,^14^ while others have sought to outline the inherent difficulties associated with quantitative PFM^15^,^16^,^17^,^18^. Inhomogeneous fields; the small volume of material excited, which is clamped by the surrounding unexcited material; choice of cantilever, that is, spring constant, tip coating and uncertainties about the tip-surface contact; and electrostatic effects all lead to low confidence in PFM as a quantitative tool. Despite these obstacles, we can minimize these experimental issues through several simple precautions. Measurements were performed on the same sample over many wires and carried out on the same day with the same cantilever and same laser spot position on the cantilever backside (known to affect measurement^15^) and with calibration of the photodiode sensitivity. Tapping mode was used to locate the nanowire and helped preserve the tip shape while contact mode was only used for point measurement; no contact scanning was performed during the course of *d*_33_ experiments. Electrostatic effects are thought to be small due to the relatively stiff cantilever used (∼1–3 Nm^−1^) and the low biases involved (1 V or less).

**Figure 4:**
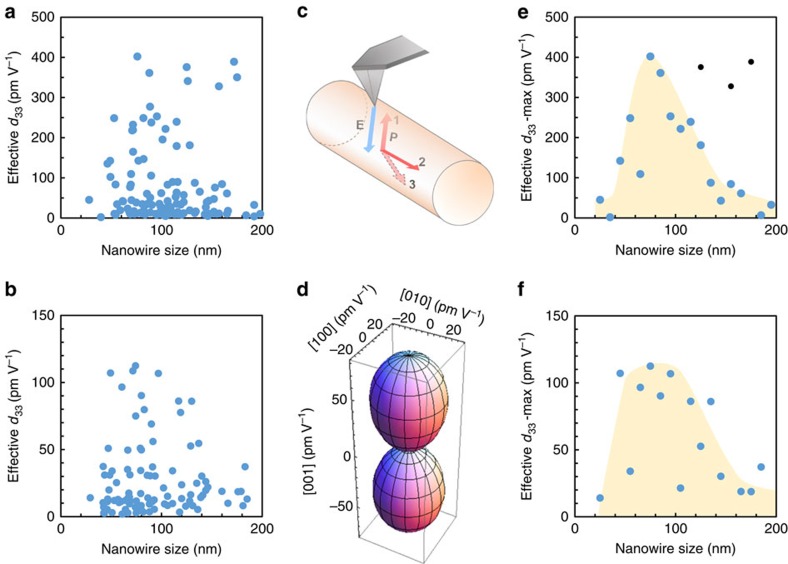
Effective *d*_33_ for PTO and PZT 13:87. Entire data set of effective *d*_33_ values measured by PFM on PTO nanowires (**a**) and on PZT 13:87 (**b**). Data collected over 24 PTO wires and 30 PZT 13:87 wires. (**c**) Schematic of a nanowire with the orientation of its spontaneous polarization aligned with the applied field **E** emanating from the tip, state (1), perpendicular to the tip (2) or at some intermediate angle between the two (3). (**d**) *d*_33_ of PTO at 25 °C as a function of the angle between the orientation of the spontaneous polarization and the applied electric field calculated using theoretical *d*_33_, *d*_31_ and *d*_15_ of ref. 25. The diagram is plotted in the spherical coordinates system, and the vertical axis is parallel to the direction of the spontaneous polarization. It is seen that the measured *d*_33_ is equal to the true *d*_33_ only when the direction of the spontaneous polarization is parallel to the applied field. *d*_33_-max (the maximum value of *d*_33_ obtained for wires within a moving window of 10 nm) as a function of wire diameter for PTO (**e**) and PZT 13:87 (**f**). The handsketched yellow background in **e** and **f** is to highlight the envelope of *d*_33_-max as a guide to the eye. In **e** the anomalous points (black) have been excluded from the maximum values but are plotted for clarity.

In the microscope used (Asylum Research Cypher), a resonance technique is generally used for PFM called dual AC resonance tracking or DART for short^19^. This mode utilizes the electromechanical resonance of the combined cantilever–piezoelectric system and thus allows measurements of ferroelectric and piezoelectric materials with low piezoelectric coefficients, that is <10 pm V^−1^. To obtain the surface deformation of the piezoelectric material due to the application of a voltage we can model the cantilever-sample system as a driven damped harmonic oscillator according to refs 20, 21 to give the measured piezoresponse amplitude as a function of driving frequency, *f*, so that


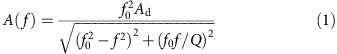


where *A*_d_ is the amplitude of the electric-field-induced surface deformation driving the system, *f*_0_ is the resonant frequency and *Q* is the quality factor. As the DART mode uses two excitation frequencies *f*_1_, *f*_2_ this leads to four measured quantities, namely the corresponding amplitudes *A*_1_, *A*_2_ and phases *φ*_1_, *φ*_2_. This is sufficient to obtain values for *Q* and *f*_0_ from which *A*_d_ is calculated. This procedure, according to the method outlined in ref. 20, is performed by the Asylum Research software (Version 13.16.100). Furthermore, since *A*_d_=*d*_33_*V*_ac_, we can obtain values for the *d*_33_ piezoelectric coefficient. A range of nanowires of various sizes was selected for investigation and several points per nanowire were measured. The entire data set for PTO is shown in Fig. 4a, and that for the PZT nanowires in Fig. 4b.

Clearly the measured *d*_33_ values are scattered. This is not surprising, taking into account the randomness of the orientation of the *c* axis (orientation of the spontaneous polarization) relative to the direction of the applied electric field (Fig. 4c). While the electric field applied from the metallic tip is perpendicular to the substrate plane, the orientation of the polar axis relative to the longitudinal axis of the nanowires is uncontrolled during their preparation and dispersion on the substrate.

The angle between the orientation of the spontaneous polarization and the applied electric field strongly influences the measured *d*_33_, as shown in Fig. 4d using theory. The measured *d*_33_ deviates therefore from the real longitudinal *d*_33_. The inclination angle of the spontaneous polarization from the applied electric field varies from wire to wire and from domain to domain along each nanowire. Therefore the entire data set of the measured *d*_33_ can show values that scatter between 0 and the real longitudinal *d*_33_. The above consideration indicates also that the highest measured *d*_33_ values within a statistically meaningful collected data represent domains in the nanowire with polarization orientation nearest to the orientation that would give the real *d*_33_. In addition, extra data sets obtained in different experimental sessions support the trend and give further support (Supplementary Fig. 2) that the maximum measured *d*_33_ is related to the case of alignment of the polarization with the applied electric field as seen in Fig. 4c case (1). We apply this consideration on Fig. 4a and draw in Fig. 4e the *d*_33_-max (the maximum value of *d*_33_ obtained for wires within a moving window of 10 nm, for example, 11–20, 21–30 nm and so on) as a function of wire diameter. Some three- to fourfold enhancement of *d*_33_ of the most active wires (∼100 nm) can be observed, both relative to the very thin wires and relative to the known *d*_33_ value of bulk PTO (∼80 pm V^−1^). The same is done for the PZT nanowires, presented in Fig. 4f. In the PZT, the change in *d*_33_-max as a function of the wire diameter is weaker but still present, which is consistent with the less-pronounced enhancement of *c*/*a* as shown in Fig. 2c. It seems therefore that the negative pressure results in enhancement of the piezoelectric response.

### Mesoscopic simulation of piezoresponse in nanowires

**Figure 5:**
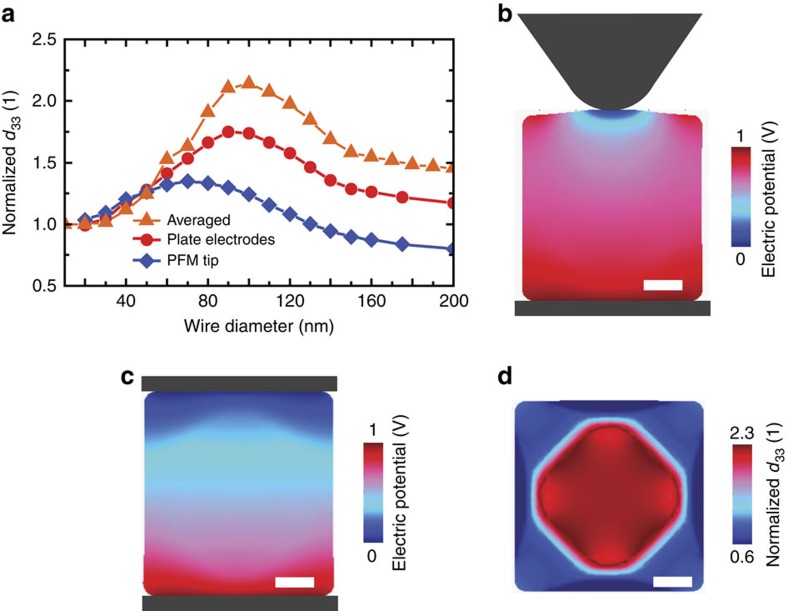
Numerically simulated piezoresponse of nanowires. (**a)** Effective *d*_33_ (normalized with respect to the bulk *d*_33_ at zero pressure) is plotted as a function of wire size for three cases. Blue diamonds in **a** correspond to the wire's mechanical response to a static electrical excitation with a ∼50 nm diameter PFM tip. The corresponding distribution of the electric potential is shown in a colour-scale map, in **b**. Red circles in **a** are calculated from the wire's response to the electric field between two planar electrodes. Corresponding potential distribution is shown in **c**. Orange triangles in **a** result from *d*_33_ averaging over the negative pressure region, which suggests maximal theoretically achievable enhancement of piezoelectric properties. The distribution of normalized *d*_33_ is shown in **d**. Scale bar in **b**, **c** and **d**, 20 nm.

To link the first principles results that describe the bulk properties of ferroelectrics with the experimental PFM results on actual nanowires, we developed a field model, which numerically simulates the effective piezoelectric response of inhomogeneously stressed nanowires. The inhomogeneous distribution of hydrostatic pressure in real nanowires is a consequence of the gradual inward phase transformation from a lower- to higher-density phase^6^. This transformation is accompanied with non-elastic deformations, which finally develop negative pressure in the inner part of a wire at the expense of a surface shell that is under compressive stresses. Close analogy can be seen in the solidification of glasses^22^. The transformed nanowire is therefore a composite of regions with different properties. For this purpose we modified and upgraded the 2D elasto-plastic model of the PTO nanowire developed in ref. 6. The model is solved in two steps. First, the phase transformation is simulated for each wire diameter as in ref. 6, but with parameters modified to fit the experimentally observed *c*/*a* ratio with the new first principles data presented in this work. The obtained pressure distribution is linked to distribution of piezoelectric coefficients via the first principles data shown in Fig. 1a. In the second step, the piezoelectric response is calculated with the conventional piezoelectric materials module in Comsol 4.3a. Three configurations are analysed as shown in Fig. 5. For the first configuration, the effective *d*_33_ coefficient is calculated from the displacement of a nanowire that is excited by an electric field between a bottom electrode and an ∼50-nm diameter PFM tip (Fig. 5b). For the second configuration the nanowire is assumed between planar electrodes (Fig. 5c). The third configuration is given for comparison, here *d*_33_ is averaged over the negative pressure region, Fig. 5d, which suggests maximal achievable enhancement of the piezoelectric properties. The polar axis in all three cases is oriented up as in the Fig. 4c (along the electric field). Qualitatively, the model is valid also for PZT and BTO nanowires. Note that the *d*_33_ drop above ∼100 nm nanowire diameters may—in experiments—occur from two different reasons: due to the drop of material *d*_33_ at negative pressures above 1 GPa or due to the pressure release after cavitation processes or cracking^6^. The simulation covers only the former scenario but the *d*_33_ trend is expected coincidently to be similar in both cases. The piezoelectric model considers constant elastic stiffness and permittivity in the whole nanowire, but the inhomogeneous distribution of the piezoelectric properties causes the nanowire core being mechanically clamped by its less piezoelectrically active outer shell during electrical excitation as seen in Supplementary Fig. 3. Nevertheless, the model shows that the pronounced enhancement of piezoelectric properties should be clearly measurable in the PFM experiment. Simulation details are described in Methods and Supplementary Note 1.

## Discussion

The *ab initio* calculation shows an enhancement with negative pressure, where uniform stress is applied. However, PFM is conducted on nanowires of which the build-in stress is not strictly uniform. In addition to that the electric field distribution is not uniform. The numerical simulations link the *ab initio* results with the PFM data and also act as a bridge between the two. The simulated PFM data correlate well with the wire diameter at which the peak response is found from the real-world PFM experiments. This increases the confidence that the negative pressure is in fact responsible for enhancement of piezoelectricity and should not only be applicable to the materials under investigation here but also to other cases with suitable geometries and phase transitions, for example, ref. 23. Indeed, the numerical simulations can provide a route towards optimization of geometries that are amenable to negative pressure generation and exploration of how resulting material parameters will therefore be affected.

In summary, first principles calculations show a substantial enhancement of longitudinal and hydrostatic piezoelectric coefficients under negative pressure, in lead titanate, PZT and barium titanate, associated with the tetragonal to super-tetragonal evolution of the studied systems. In the case of PTO and PZT, the smooth evolution is governed by crossing of a supercritical trace while BTO undergoes an isomorphous first-order phase transition, which results in discontinuity of the piezoelectric coefficients. The increase in the longitudinal piezoelectric coefficient on increase in negative pressure was detected also experimentally in PTO and PZT and agreement exists between the computed and measured results. Numerical simulations confirm that negative pressure is present in the wire and is the origin of the enhanced piezoelectric coefficients measured by experiment. The obtained results point therefore to a new direction for possible enhancements in piezoelectric properties of commonly used ferroelectric perovskites.

## Methods

### *Ab inito* calculations

*Ab initio* calculations were performed using the Quantum ESPRESSO package^24^ within the generalized gradient approximation with PBESOL pseudopotentials from PSLibrary 0.3.1(qe-forge.org/gf/project/pslibrary). Using PWscf code, we performed full relation ground-state energy calculations under different hydrostatic pressures. The parameters of the calculations were the following: the kinetic energy cutoff for wavefunctions was 60 Ry and the energy cutoff for electronic density was 760 Ry. We used an automatically generated uniform Monkhorst–Pack *k*-points grid of 8 × 8 × 8 points for tetragonal cells of PTO and BTO and 4 × 4 × 4 points for PZT. The convergence threshold for ionic minimization on residual Hellmann–Feynman forces was 10^−5^ a.u. (2 meV A^−1^). The threshold on the residual pressure was 0.2 kBar. For calculations of the PZT solid solution, we constructed a 2 × 2 × 2 supercell containing one cell of PbZrO_3_ and seven cells of PTO. This way we replace a real PZT material (with a random distribution of the Zr and Ti atoms) by a regular structure with a periodic distribution of atoms. This approximation takes into account the impact of the average Zr concentration, neglecting that of its spatial concentration fluctuations. To have an idea about the inaccuracy associated with such neglect, we evaluated *d*_33_ for a 2 × 2 × 4 supercell containing two cells of PbZrO_3_ in neighbouring positions, corresponding to the case of the strongest possible fluctuation of the Zr concentration. This leads to a value of 106 versus 100 pC N^−1^ for the 2 × 2 × 2 supercell calculations. This suggests a reasonable accuracy of our 2 × 2 × 2 supercell calculations.

Polarization was calculated by atomic displacements multiplied by the Born charges. The calculated Born charges along the polar *c* axis were *Z*_Pb_=3.6, *Z*_Ti_=4.7, *Z*_O1_=−2.0, *Z*_O2_=−4.1 for PTO; *Z*_Pb_=3.7, *Z*_Zr_=6.0, *Z*_Ti_=4.7, *Z*_O1_=−2.1, *Z*_O2_=−4.2 for PZT; and *Z*_Ba_=2.7, *Z*_Ti_=7.4, *Z*_O1_=−2.0, *Z*_O2_=−5.8 for BTO.

### Piezoresponse force microscopy

PFM was performed by an Asylum Research Cypher atomic force microscope operating in DART mode. ASYELEC-01 probes of force constant ∼1–3 Nm^−1^ and a conductive coating of Ti/Ir were used. The radius of curvature for these probes is ∼50 nm.

### Mesoscopic simulation

Piezoresponse of nanowires was calculated in two steps. First, the process of negative-pressure formation during the structural phase transition from PX to the perovskite phase was simulated with a 2D numerical model of an elasto-plastic material whose spontaneous strain is coupled (through a phase parameter) with the concentration of diffusing catalytic oxygen. The simulation domain has a square shape of varying size with round fillets. The boundary conditions are set as stress-free with the influx of oxygen. The phase change is defined as a change of spontaneous strain at a specified oxygen concentration. The model is implemented in the finite-element method software COMSOL 4.3a by modification of its standard heat transfer and solid mechanics modules. Second, the distribution of *d*_33_ in the nanowire was associated with the negative pressure via the first principles calculated data. The effective static piezoresponse was calculated as a mechanical displacement per applied electric potential at two introduced types of electrodes. Model was solved with the standard piezoelectric material module in Comsol 4.3a. More detailed descriptions on the methods can be found in Supplementary Note 1.

### Data availability

The data supporting the findings of this study are available from the corresponding author on request.

## Additional information

**How to cite this article:** Kvasov, A. *et al.* Piezoelectric enhancement under negative pressure. *Nat. Commun.* 7:12136 doi: 10.1038/ncomms12136 (2016).

## Supplementary Material

Supplementary Information Supplementary Figures 1-3, Supplementary Note 1 and Supplementary References
