# Supplementary material for: Piezoelectric enhancement under negative pressure
Source: Nat Commun. 2016 Jul 11;7:12136. doi: 10.1038/ncomms12136 (PMC4942569; doi:10.1038/ncomms12136)
Supplement: Supplementary Information — Supplementary Figures 1-3, Supplementary Note 1 and Supplementary References [file ncomms12136-s1.pdf]

Supplementary Figures

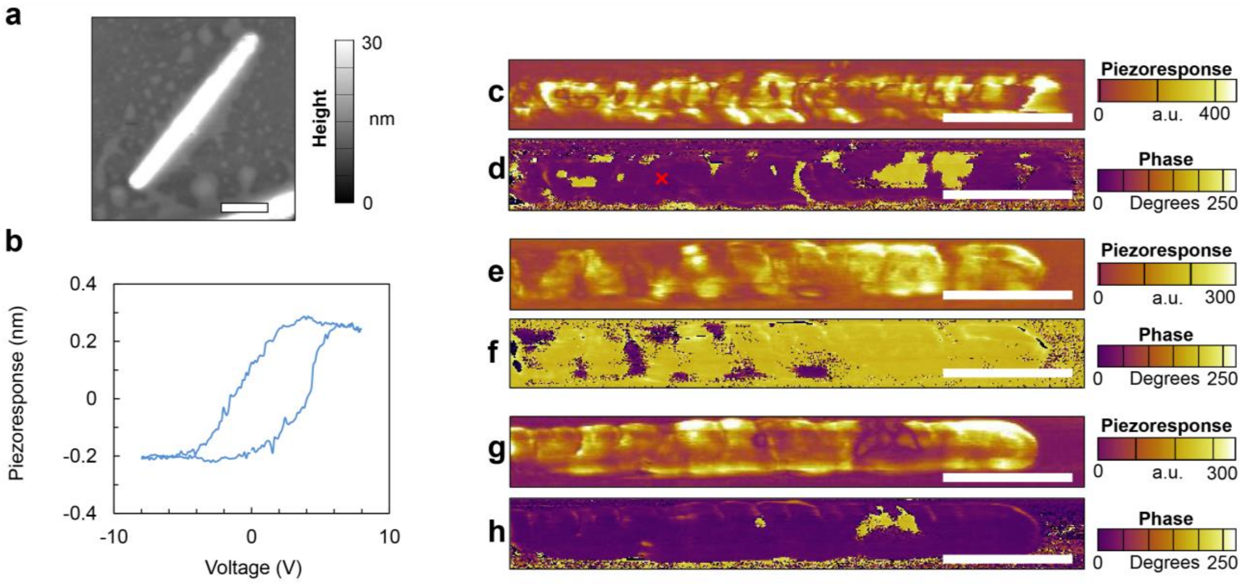

Supplementary Figure 1. PFM and switching of an individual PZT nanowire. (**a**) Topography image of a PZT nanowire. (**b**) Hysteresis loop obtained in the position of the red cross in (**d**). PFM amplitude (**c**) and phase (**d**) of the native multidomain structure and after scanning with +5 V [amplitude: (**e**) and phase: (**f**)] and -5 V applied to the tip [amplitude: (**g**) and phase: (**h**)]. All scale bars are 200 nm.

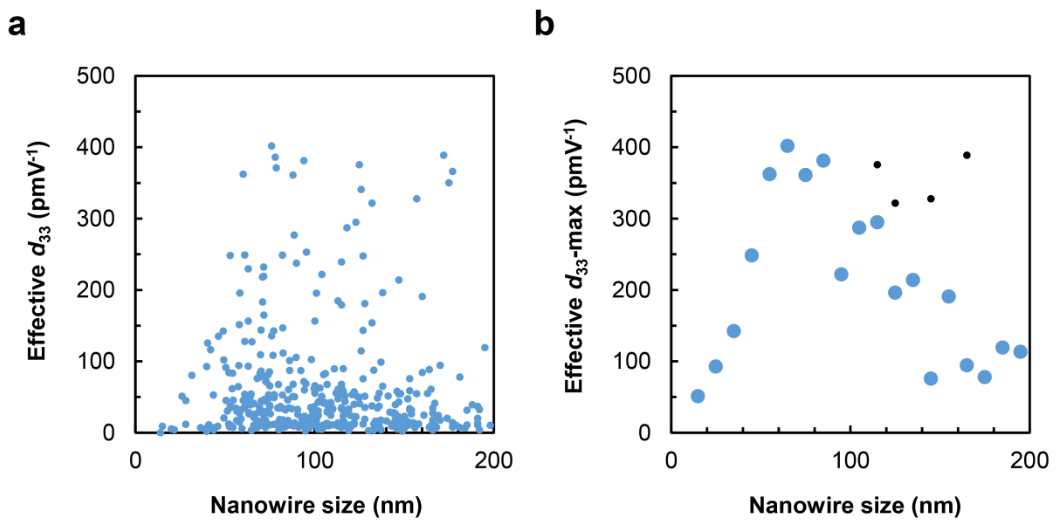

Supplementary Figure 2. PFM measurement of effective piezoelectric coefficient. Total experimental data with 450 data points on 97 individual PTO nanowires (**a**) and the maximum values of d_{33} in a 10 nm moving window (**b**). The black points are anomalous but are plotted for clarity.

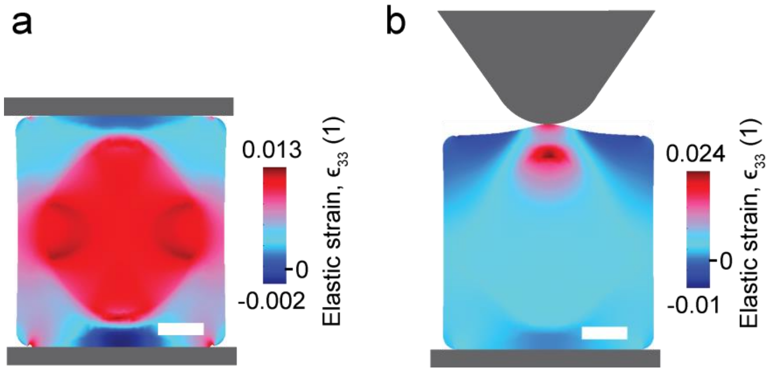

Supplementary Figure 3. **Elastic strain in nanowires**. Distribution of the elastic strain component ϵ_{33} (polar axis points up in the image) produced by the piezoelectric effect of the nanowire between planar electrodes (a) demonstrates the effect of the nanowire clamping by its outer shell with smaller piezoelectric coefficient (-1V at the top electrode, ground at the bottom electrode). The piezoelectric effect produces significantly larger strain in the inner region with the enhanced piezoelectricity than in the outer shell. The total displacement of the nanowire is therefore hindered by the less piezoelectrically strained outer shell. When a PFM tip is used as an electrode, (b), one can see that only limited surface region is prober. In this case, the effective piezoelectric response of the nanowire depends on the ratio between the tip diameter and the thickness of the outer shell. Only when the ratio is less than 1, the effective piezoelectric response measured by the PFM technique is almost entirely given by the piezoelectric properties of the outer shell which is however not the case in the investigated situation. The scale bars are 20 nm.

Supplementary Notes

Supplementary Note 1. Mesoscopic simulation of nanowire piezoelectric response

In order to obtain the effective piezoelectric response of a nanowire in simulated experimental conditions, we have to find first the stress distribution inside the nanowire. We follow the approach used in Ref. 1, relying now on the updated first principles data on *c/a* ratio vs. pressure. To fit the experimental results with the new first principles data, we have to change the parameter which controls the phase boundary thickness.

We use a 2D plane-strain elasto-plastic model which is combined with a phase transformation that proceeds from the surface inwards and induces gradual volume shrinkage. The phase, described with a parameter $\varphi \in (0,1)$ where 0 stands for pure PX and 1 for pure perovskite phase, is a function of the actual concentration C of the catalytic oxygen. The oxygen diffuses relatively slowly from the surface of the nanowire to its volume which is the reason for the gradual inward phase transformation. The evolution of concentration C is calculated with a classical diffusion equation $\frac{\partial C}{\partial t} = \nabla.(D\nabla C)$ and with boundary conditions assuming oxygen influx to the wire in analogy with the heat surface radiation $\mathbf{n}.(-D\nabla C) = e\,C^4$. Here D is a diffusion coefficient, $\mathbf{n}$ is the surface normal vector and e is the oxygen transmissibility through the surface. D and e determine the speed of oxygen influx and spatio-temporal evolution of its concentration C. The initial oxygen concentration inside the wire is set slightly below the defined phase transition point C_0, namely C_0/C=1.0017, to minimize calculation time before the phase transformation starts.

The phase transition occurs at the location where $C = C_0$. The phase boundary, however, is not abrupt. It has a smooth profile given by $\varphi = (1 - \tanh(\gamma\,(C_0 - C)))/2$, where γ is a constant determining the width of a phase boundary together with the gradient of oxygen concentration. The change of the phase parameter φ from 0 to 1 is accompanied with 13% volume reduction which is introduced to the elasto-plastic model below through the change of diagonal components of the spontaneous-strain $\epsilon_{0,ij} = -a\,\varphi(T)$ for i=j=1,2, when a=0.063 (in case of a 2D model).

The equations of the elasto-plastic model assume a bilinear stress-strain relation and the isotropic hardening as implemented in the software Comsol Multiphysics. In this model, the constitutive relation $\mathbf{S} = \mathbf{C}{:}\,\boldsymbol{\epsilon}_{\mathrm{el}}$ is expressed in the form with the Green-Lagrange elastic strain tensor $\boldsymbol{\epsilon}_{\mathrm{el}} = \boldsymbol{\epsilon}_{\mathrm{t}} - \boldsymbol{\epsilon}_0 - \boldsymbol{\epsilon}_{\mathrm{p}}$ and the second Piola-Kirchhoff stress tensor **S**. Elastic stiffness tensor **C** is expressed classically in terms of Young's modulus E and Poison's ratio ν introduced below. The total Green-Lagrange strain $\boldsymbol{\epsilon}_{\mathrm{t}}$ is defined as $\boldsymbol{\epsilon}_{\mathrm{t}} = \frac{1}{2}(\mathbf{F}^{\mathbf{T}}\mathbf{F} - \mathbf{I})$ where $\mathbf{F} = \mathbf{I} + \nabla\mathbf{u}$ is the deformation gradient in which $\mathbf{u}$ is a displacement vector of material. $\boldsymbol{\epsilon}_0$ is the volume-shrinkage strain introduced above as the consequence of the phase transition. The plastic strain $\boldsymbol{\epsilon}_p$ is integrated from increments

$d\boldsymbol{\epsilon}_{\mathrm{p}} \sim \frac{\partial F_{\mathrm{y}}}{\partial \boldsymbol{\sigma}}\, d\boldsymbol{\sigma}$ where $F_{\mathrm{y}} = \sigma_{\mathrm{mises}}(\boldsymbol{\sigma}) - \sigma_{\mathrm{ys}} \leq 0$ is the yield criterion and $\boldsymbol{\sigma} = \det(\mathbf{F})^{-1}\mathbf{FSF}^{\mathrm{T}}$ the Cauchy stress. Here, σ_{mises} is the von Mises stress and $\sigma_{\mathrm{ys}} = \sigma_{\mathrm{ys0}} + \frac{E_{\mathrm{Tiso}}}{1-\frac{E_{\mathrm{Tiso}}}{E}}\, \epsilon_{\mathrm{pe}}$ is the yield stress where σ_{ys0} is the initial yield stress, E_{Tiso} is the isotropic tangent modulus in the plastic regime, and ϵ_{pe} is the corresponding component of the effective (i.e. accumulated) plastic strain. The stress-strain distribution during the phase transition with volume shrinkage due to $\boldsymbol{\epsilon}_0$ must satisfy the stress equilibrium $-\nabla.\boldsymbol{\sigma} = \mathbf{0}$ where inertia and volume forces are neglected.

Note, that the exact physics and parameter values of the oxygen diffusion, phase transformation and the plastic deformation are unknown. Therefore, we have chosen the simplest models in order to demonstrate the fundamental mechanism and we used the values of unknown model parameters to fit the experimentally observed *c/a* ratio at specific wire diameters.

The introduced model equations are solved on a square geometry with round fillets on its edges (to minimize numerical errors due to singularities). The simulation results were evaluated for a set of wire diameters from 10 to 200 nm with parameters $a = 0.063$, $\sigma_{\mathrm{ys0}} = 1.2\ 10^{10}$ Pa, $E_{\mathrm{Tiso}} = 10^{11}$ Pa, $E = 4.5\ 10^{11}$, $\nu = 0.2$, $D = 2\ 10^{-11} \mathrm{m}^2/\mathrm{s}$, $e = 2\ 10^{-15}$, $C_0 = 600\ \mathrm{mol/m}^3$, $\gamma = 6\ \mathrm{m}^3/\mathrm{mol}$. Note that the parameter γ is different from the one used in Ref. 1.

Numerical solution of the above introduced elasto-plastic model gives the stress and strain distribution in each transformed nanowire.

In the second step of the model we evaluate the distribution of the hydrostatic pressure inside the nanowires and define the local relative d_{33} enhancement via the first principles data displayed in Fig. 1a. The absolute values of the material data with the polar axis oriented along y-coordinate correspond to the material PZT-5J from the standard Comsol library. The relative macroscopic enhancement of piezoelectricity, which we evaluate, is however almost independent on the absolute values of material parameters.

The nanowire is mechanically clamped on the bottom (*y=0*) boundary and electrodes are defined on the bottom (*y=0*) and top (*y*=wire width) surfaces of the nanowire. Remaining surfaces are stress-free and with zero free charge. First, electrodes are defined as planar and the piezoresponse is calculated as the maximal mechanical displacement at applied potential difference of 1 V (Fig. 5c). Second, the potential distribution on the top electrode is given by a Gaussian distribution with standard deviation 10 nm and amplitude 1 V. This distribution mimics the potential under approx.50 nm scanning probe tip. Piezoresponse is evaluated from the maximal mechanical displacement under the PFM tip at 1 V (Fig. 5b). Last, the d_{33} coefficient is averaged over the central region under the negative pressure (Fig. 5d).

All three types of d_{33} evaluation in Fig. 5 show a peak value at wire diameters where *c/a* ratio undergoes abrupt growth as seen experimentally and as fitted by the model. However, the reason for the sudden drop of d_{33} above these peaks may occur from two different reasons. First, due to the drop of material d_{33} at higher negative pressures as seen in first principles calculation or, second, due to the release of negative pressure originating from experimentally observed nanopore formation or cracking. Obviously, the introduced model cannot predict the latter scenario as it does not incorporate any stress release mechanisms like cavitation process and a finite fracture toughness, but the final impact on d_{33} trend (unlike on *c/a* trend) remains coincidentally similar.

The effect of nanowire clamping by its outer shell is well seen on the strain distribution under the electric field in Supplementary Fig. 3.

Supplementary References

1. Wang, J. *et al.* Negative-pressure-induced enhancement in a freestanding ferroelectric, *Nat. Mat.* **14,** 985-990 (2015).
